# Receptor protein tyrosine phosphatase beta/zeta is a functional binding partner for vascular endothelial growth factor

**DOI:** 10.1186/s12943-015-0287-3

**Published:** 2015-02-03

**Authors:** Marina Koutsioumpa, Evangelia Poimenidi, Evangelia Pantazaka, Christina Theodoropoulou, Angeliki Skoura, Vasileios Megalooikonomou, Nelly Kieffer, Jose Courty, Shuji Mizumoto, Kazuyuki Sugahara, Evangelia Papadimitriou

**Affiliations:** Laboratory of Molecular Pharmacology, Department of Pharmacy, University of Patras, GR 26504 Patras, Greece; Computer Engineering and Informatics Department, University of Patras, GR 26504 Patras, Greece; Sino-French Research Centre for Life Sciences and Genomics, CNRS/LIA124, Rui Jin Hospital, Jiao Tong University Medical School, Shanghai, China; Laboratoire CRRET, Universite Paris Est Creteil Val de Marne, Paris, France; Proteoglycan Signaling and Therapeutics Research Group, Faculty of Advanced Life Science, Hokkaido University, Sapporo, Japan; Current address: Center for Systems Biomedicine, Division of Digestive Diseases, David Geffen School of Medicine, University of California Los Angeles, Los Angeles, CA USA; Current address: Department of Pathobiochemistry, Faculty of Pharmacy, Meijo University, Nagoya, 463-8503 Japan

**Keywords:** Chondroitin sulphate, Endothelial cells, Migration, Pleiotrophin, Tyrosine phosphatases, Vascular endothelial growth factor

## Abstract

**Background:**

Receptor protein tyrosine phosphatase beta/zeta (RPTPβ/ζ) is a chondroitin sulphate (CS) transmembrane protein tyrosine phosphatase and is a receptor for pleiotrophin (PTN). RPTPβ/ζ interacts with α_ν_β_3_ on the cell surface and upon binding of PTN leads to c-Src dephosphorylation at Tyr530, β_3_ Tyr773 phosphorylation, cell surface nucleolin (NCL) localization and stimulation of cell migration. c-Src-mediated β_3_ Tyr773 phosphorylation is also observed after vascular endothelial growth factor 165 (VEGF_165_) stimulation of endothelial cells and is essential for VEGF receptor type 2 (VEGFR2) - α_ν_β_3_ integrin association and subsequent signaling. In the present work, we studied whether RPTPβ/ζ mediates angiogenic actions of VEGF.

**Methods:**

Human umbilical vein endothelial, human glioma U87MG and stably transfected Chinese hamster ovary cells expressing different β_3_ subunits were used. Protein-protein interactions were studied by a combination of immunoprecipitation/Western blot, immunofluorescence and proximity ligation assays, properly quantified as needed. RPTPβ/ζ expression was down-regulated using small interference RNA technology. Migration assays were performed in 24-well microchemotaxis chambers, using uncoated polycarbonate membranes with 8 μm pores.

**Results:**

RPTPβ/ζ mediates VEGF_165_-induced c-Src-dependent β_3_ Tyr773 phosphorylation, which is required for VEGFR2-α_ν_β_3_ interaction and the downstream activation of phosphatidylinositol 3-kinase (PI3K) and cell surface NCL localization. RPTPβ/ζ directly interacts with VEGF_165_, and this interaction is not affected by bevacizumab, while it is interrupted by both CS-E and PTN. Down-regulation of RPTPβ/ζ by siRNA or administration of exogenous CS-E abolishes VEGF_165_-induced endothelial cell migration, while PTN inhibits the migratory effect of VEGF_165_ to the levels of its own effect.

**Conclusions:**

These data identify RPTPβ/ζ as a cell membrane binding partner for VEGF that regulates angiogenic functions of endothelial cells and suggest that it warrants further validation as a potential target for development of additive or alternative anti-VEGF therapies.

**Electronic supplementary material:**

The online version of this article (doi:10.1186/s12943-015-0287-3) contains supplementary material, which is available to authorized users.

## Background

Vascular endothelial growth factor A (VEGF) is a growth factor that activates several functions of endothelial cells, thus triggering angiogenesis and vascular permeability. It exists as different isoforms of 121, 145, 165, 189 and 206 amino acids, among which VEGF_165_ is dominant in terms of amount and biological activity. VEGF_165_ is over-expressed in a variety of human tumors, and its over-expression is correlated with progression, invasion, and metastasis of tumors. VEGF_165_ cell signaling leading to increased endothelial cell migration and tubular formation is mediated via vascular endothelial growth factor receptor 2 (VEGFR2), which interacts with co-receptors, such as α_ν_β_3_ integrin [1]. It has been shown that c-Src-mediated phosphorylation of β_3_ cytoplasmic tail tyrosine residues occurs in response to VEGF_165_ and is essential for VEGFR2-β_3_ integrin association and subsequent signaling [2,3]. Up to date, it remains unclear how c-Src is activated by VEGF_165_. The first step in c-Src activation requires dephosphorylation of its carboxy-terminus Tyr530 [4], suggesting that a tyrosine phosphatase may be involved.

Receptor protein tyrosine phosphatase beta/zeta (RPTPβ/ζ) is a member of the family of receptor-type transmembrane protein tyrosine phosphatases that interacts with several cell adhesion molecules, such as neutral cell adhesion molecule, neuron-glia cell adhesion molecule, tenascin and contactin. It also acts as a receptor for the soluble, heparin-binding growth factors midkine and pleiotrophin (PTN) [5]. RPTPβ/ζ and PTN are expressed in endothelial cells [6] and over-expressed in several types of cancer [5,7]. PTN binding to RPTPβ/ζ on endothelial cells leads to Tyr530 dephosphorylation and activation of c-Src, β_3_ Tyr773 phosphorylation, and PTN-induced endothelial cell migration and tube formation on matrigel [5,8]. Besides PTN, RPTPβ/ζ has been shown to be the putative receptor for the vacuolating cytotoxin VacA produced by *Helicobacter pylori* [9], as well as a functional receptor for interleukin-34 [10], suggesting that it acts as a functional binding partner for several soluble molecules.

We have recently shown that RPTPβ/ζ-induced, c-Src-mediated β_3_ Tyr773 phosphorylation is also required for PTN-induced cell surface nucleolin (NCL) localization [11]. NCL is over-expressed on the plasma membrane of cancer and activated endothelial cells and has been shown to play critical roles in the modulation of tumorigenesis and angiogenesis through its interaction with a variety of ligands, among which tumor homing peptide F3, endostatin, P-selectin and PTN [12]. VEGF_165_ induces NCL localization on the surface of endothelial cells and this effect is considered important for its angiogenic actions [13,14]; however, the receptors and pathways involved have not been elucidated.

In the present work, we explored the possibility that RPTPβ/ζ is involved in the stimulatory effect of VEGF_165_ on endothelial cell signaling leading to cell migration. Our data show that VEGF_165_ directly interacts with RPTPβ/ζ to induce c-Src-mediated β_3_ Tyr773 phosphorylation. The latter is required for both cell surface NCL localization and increased interaction of α_ν_β_3_ with VEGFR2, leading to VEGF_165_-induced endothelial cell migration.

## Results and discussion

### Phosphorylation of β_3_ Tyr773 is required for VEGF_165_-induced cell migration and cell surface NCL localization

**Figure 1:**
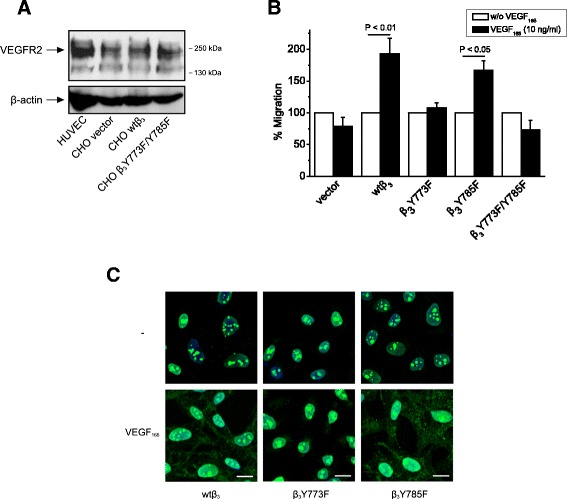
**Phosphorylation of β**_**3**_ **Tyr773 is required for VEGF**_**165**_ **-induced cell migration and cell surface NCL localization. (A)** Protein extracts of CHO cells were analysed for expression of VEGFR2. HUVEC were used as a positive control and β-actin as a loading control. **(B)** Effect of VEGF_165_ (10 ng/ml) on CHO cell migration. Data are from five independent experiments and are expressed as mean ± s.e.m. percentage change in number of migrating cells compared with the corresponding non stimulated cells (set as default 100). **(C)** Immunofluorescence images stained for NCL (green) and nucleus (blue) in serum starved CHO cells treated with VEGF_165_ (10 ng/ml) for 5 h at 37°C. Vector, cells transfected with the plasmid vector; wtβ_3_, cells over-expressing wild-type β_3_; β_3_Y773F, cells over-expressing β_3_Y773F; β_3_Y785F, cells over-expressing β_3_Y785F; β_3_Y773F/Y785F, cells over-expressing double mutant β_3_Y773F/Y785F.

It has been shown that phosphorylation of β_3_ cytoplasmic Tyr 773 and 785 in response to VEGF_165_ plays a role in endothelial cell migration [2]. In order to determine which of the two Tyr is responsible for VEGF_165_-induced cell migration, we used CHO cells that express VEGFR2 (Figure 1A), RPTPβ/ζ and α_ν_ [8,11], but do not express β_3_ and are mock-transfected or stably transfected to over-express wild-type β_3_ or β_3_ in which Tyr773 and/or Tyr785 are mutated to Phe [11]. VEGF_165_ induced migration of CHO cells over-expressing wild type β_3_ or β_3_Y785F, but had no effect on CHO cells over-expressing β_3_Y773F or β_3_Y773F/Y785F (Figure 1B), suggesting that β_3_ Tyr773 is important for VEGF_165_-induced cell migration. In the same line and similarly to what we have recently shown for PTN [11], VEGF_165_-induced cell surface NCL localization was only observed in CHO cells over-expressing wild type-β_3_ or β_3_Y785F, while in cells over-expressing β_3_Y773F, NCL remained restricted in the cell nucleus, suggesting that β_3_ Tyr773 but not Tyr785 phosphorylation is important for VEGF_165_-induced cell surface NCL localization (Figure 1C). Since RPTPβ/ζ is involved in PTN-induced β_3_ Tyr773 phosphorylation and cell surface NCL localization [8,11], these data lead to the hypothesis that RPTPβ/ζ may also be involved in VEGF_165_-induced signaling that leads to endothelial cell migration.

### RPTPβ/ζ plays a role in VEGF_165_-induced endothelial cell signaling that leads to cell surface NCL localization

**Figure 2:**
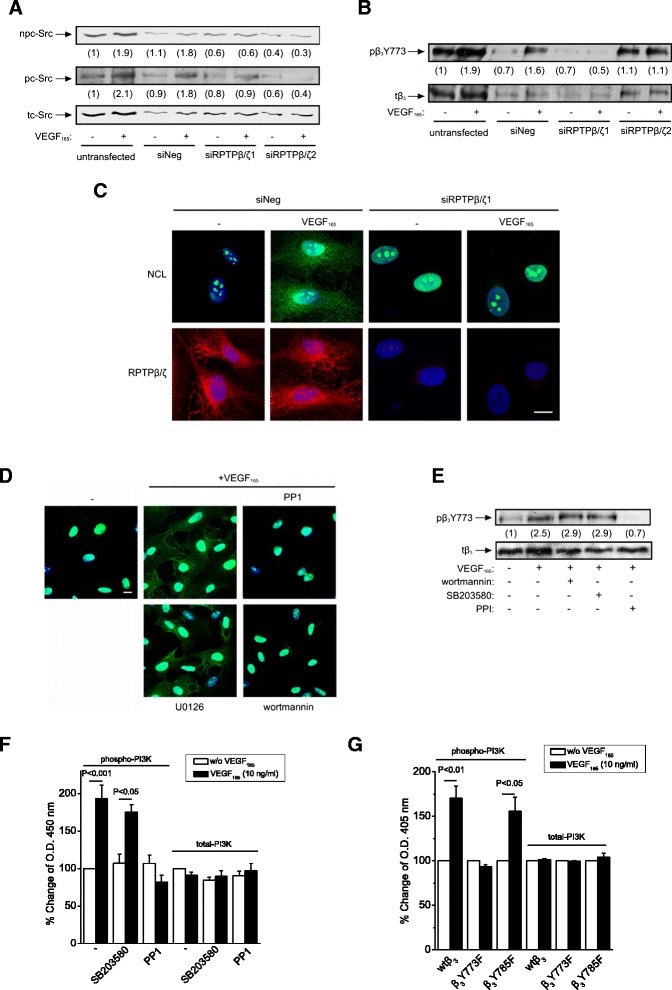
**RPTPβ/ζ is required for VEGF**_**165**_ **-induced cell surface NCL localization.** Serum starved HUVEC were treated with VEGF_165_ (10 ng/ml) for 10 min. Cell lysates were analyzed by Western blot for non Tyr530 phosphorylated (npc-Src), Tyr419 phosphorylated (pc-Src) and total (tc-Src) c-Src **(A)**, as well as for phospho-β_3_Y773 (pβ_3_Y773) and total β_3_ (tβ_3_) integrin **(B)**. Numbers in brackets denote the average-fold change of the ratio npc-Src:tc-Src, pc-Src:tc-Src or pβ_3_Y773:tβ_3_ respectively, compared with the corresponding non stimulated, untransfected cells (set as default 1). **(C)** Representative immunofluorescence images stained for NCL (green), RPTPβ/ζ (red) and nucleus (blue) from serum starved HUVEC treated with VEGF_165_ (10 ng/ml) for 5 h at 37°C. **(D)** Representative immunofluorescence images stained for NCL (green) and nucleus (blue) from serum starved VEGF_165_-stimulated HUVEC in the presence or absence of inhibitors for c-Src (PP1 10 μΜ), PI3K (wortmannin 100 nM) and ERK½ (U0126 20 nM). Scale bars in **C** and **D** correspond to 10 μm. **(E)** Lysates from serum starved VEGF_165_-stimulated HUVEC in the presence or absence of PP1 and wortmannin, were analyzed by Western blot for pβ_3_Y773 and tβ_3_ integrin. Numbers in brackets denote the average-fold change of the ratio pβ_3_Y773:tβ_3_ compared with untreated cells (set as default 1). **(F and G)** Phosphorylation of PI3K in HUVEC and CHO cells respectively. Data are expressed as mean ± s.e.m percentage change in PI3K compared with the untreated cells (set as default 100). In all cases, data come from three independent experiments. siNeg, HUVEC transfected with a negative control siRNA; siRPTPβ/ζ1, HUVEC transfected with siRPTPβ/ζ#1; siRPTPβ/ζ2, HUVEC transfected with si RPTPβ/ζ#2; vector, CHO cells transfected with the plasmid vector; wtβ_3_, CHO cells over-expressing wild-type β_3_; β_3_Y773F, CHO cells over-expressing β_3_Y773F; β_3_Y785F, CHO cells over-expressing β_3_Y785F.

Since RPTPβ/ζ is responsible for PTN-induced β_3_ Tyr773 phosphorylation through dephosphorylation and activation of c-Src in human umbilical vein endothelial cells (HUVEC) [8], we examined whether RPTPβ/ζ also affects VEGF_165_-induced c-Src activation and β_3_ Tyr773 phosphorylation. Down-regulation of RPTPβ/ζ expression by two different siRNAs abolished VEGF_165_-induced dephosphorylation of c-Src at Tyr530 (Figure 2A), suggesting that RPTPβ/ζ may be the missing link for c-Src activation upon stimulation of endothelial cells with VEGF_165_. Indeed, the increase in dephosphorylated c-Src Tyr530 was mirrored by higher phosphorylation of c-Src at Tyr419 (Figure 2A), verifying activation of c-Src. Interestingly, inhibition of VEGFR2 tyrosine kinase activity by the selective inhibitor SU1498 or inhibition of VEGF-VEGFR2 interaction by bevacizumab, did not affect VEGF_165_-induced c-Src Tyr530 dephosphorylation or c-Src Tyr419 phosphorylation (Additional file 1), suggesting that c-Src activation may be independent of VEGFR2 in these assays. RPTPβ/ζ directly interacts with c-Src [6], an observation that is in line with a role for RPTPβ/ζ in c-Src activation. Down-regulation of RPTPβ/ζ expression also abolished VEGF_165_-induced β_3_ Tyr773 phosphorylation (Figure 2B), suggesting that RPTPβ/ζ may be involved in VEGF_165_-induced signaling related to cell surface NCL localization, as has been previously described for PTN [11]. Indeed, down-regulation of RPTPβ/ζ expression abolished VEGF_165_-induced cell surface NCL localization (Figure 2C). Although it has been known for several years that VEGF_165_ induces cell surface NCL localization [13], which is required for VEGF_165_-induced cell migration [14], the receptor/pathway involved was unknown up to date. The observation that VEGF_165_-induced cell surface NCL localization depends on RPTPβ/ζ suggests a role for RPTPβ/ζ in the angiogenic effects of VEGF_165_.

To further investigate the involvement of RPTPβ/ζ in VEGF actions, we studied the role of several signaling molecules known to be activated by both VEGF_165_ and RPTPβ/ζ on cell surface NCL localization. Inhibition of c-Src and phosphatidylinositol 3-kinase (PI3K) abolished VEGF_165_-induced cell surface NCL localization, while inhibition of ERK1/2 had no effect (Figure 2D). In order to investigate whether PI3K lays up- or downstream of α_ν_β_3,_ the effect of PI3K inhibition on VEGF_165_-induced β_3_ Tyr773 phosphorylation was studied. The c-Src inhibitor PP1 was used as a positive control, since c-Src is known to lay upstream of β_3_ Tyr773 phosphorylation [2,8,11]. The p38 inhibitor (SB203580 10 μΜ, BioSource Europe, Nivelles, Belgium) was used as a negative control, since it has been previously shown not to affect RPTPβ/ζ-mediated β_3_ Tyr773 phosphorylation and PI3K activation [11]. PI3K inhibition did not affect VEGF_165_-induced β_3_ Tyr773 phosphorylation (Figure 2E), suggesting that it lays downstream of α_ν_β_3_. By using an ELISA for activated PI3K in HUVEC, it was found that inhibition of c-Src abolished VEGF_165_-induced PI3K activation (Figure 2F). Using the same assay in CHO cells over-expressing wild-type β_3_, β_3_Y773F or β_3_Y785F, it was found that VEGF_165_ significantly induced PI3K activation in CHO cells over-expressing wild-type β_3_ or β_3_Y785F, while it had no effect in cells over-expressing β_3_Y773F (Figure 2G), suggesting that PI3K lays downstream of α_ν_β_3_ and requires β_3_ Tyr773 phosphorylation. This pathway resembles the one we have recently shown for PTN-induced cell surface NCL localization [11], further supporting the notion that RPTPβ/ζ mediates this effect of VEGF_165_. Similarly to what has been previously discussed for PTN, it remains unclear how PI3K affects cell surface NCL localization. Co-immunoprecipitation of NCL with PI3K [15,16] favors a direct regulation of NCL by PI3K and one possibility is by regulating the trafficking of exocytotic vesicles. NCL has been detected in cytoplasmic vesicles fused with the plasma membrane [17], while inhibition of PI3K interferes with the trafficking of such exocytotic vesicles, affecting the number of several receptors on the plasma membrane, such as the transferrin receptor [18], the glucose transporter GLUT4 [19] or β integrin [20]. Alternatively, PI3K may indirectly regulate cell surface NCL localization, by regulating recruitment of proteins containing pleckstrin homology domains onto the cell membrane [21]. NCL does not possess such sequences but may act as a ligand for proteins containing pleckstrin homology domains through its acidic motifs [22].

### RPTPβ/ζ plays a role in VEGF_165_-induced interaction of VEGFR2 with α_ν_β_3_

**Figure 3:**
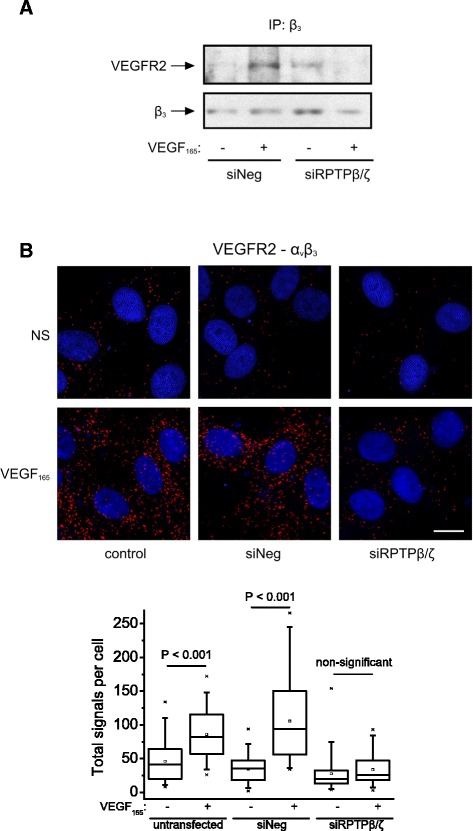
**RPTPβ/ζ regulates VEGF**_**165**_ **-induced VEGFR2-α**_**ν**_ **β**_**3**_ **interaction.** Down-regulation of RPTPβ/ζ expression by siRNA was followed by treatment of serum-starved HUVEC with VEGF_165_ (10 ng/ml) for 10 min. **(A)** Cells lysates were immunoprecipitated for β_3_ and analyzed by Western blot for the presence of VEGFR2 and β_3_. **(B)** Formation of β_3_-VEGFR2 complexes as evidenced by *in situ* PLA. The box plots indicate the median, mean and range of the detected signals (n = 8 image fields with ~4 cells per image per sample type, each sample run in duplicate). Scale bar corresponds to 10 μm. siNeg, HUVEC transfected with a negative control siRNA; siRPTPβ/ζ, HUVEC transfected with siRPTPβ/ζ#1.

Based on the literature that c-Src–mediated phosphorylation of β_3_ essentially regulates VEGF_165_-induced interaction of α_ν_β_3_ with VEGFR2 in endothelial cells [2] and our observation that RPTPβ/ζ is required for VEGF_165_-induced c-Src activation and β_3_ Tyr773 phosphorylation (Figure 2), we tested the hypothesis that RPTPβ/ζ may have a role in the interaction of α_ν_β_3_ with VEGFR2. Down-regulation of RPTPβ/ζ expression by siRNA abolished the increased interaction of α_ν_β_3_ with VEGFR2 induced by VEGF_165_, as evidenced by both immunoprecipitation/Western blot (Figure 3A) and proximity ligation assays (PLA) (Figure 3B). These data also show involvement of RPTPβ/ζ in VEGF-induced endothelial cell migration.

### RPTPβ/ζ plays a role in VEGF_165_-induced endothelial cell migration

**Figure 4:**
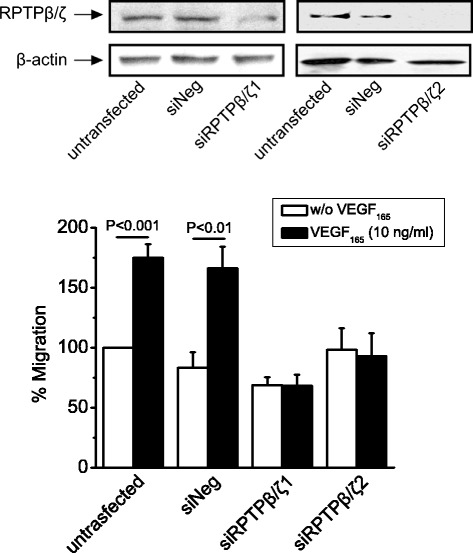
**RPTPβ/ζ is required for VEGF**_**165**_ **-induced endothelial cell migration.** Effect of VEGF_165_ on HUVEC migration after down-regulation of RPTPβ/ζ expression by two different siRNAs. Data are from five independent experiments and are expressed as mean ± s.e.m. percentage change in number of migrating cells compared with the non stimulated untransfected cells (set as default 100). Untrasfected, untransfected HUVEC; siNeg, HUVEC transfected with a negative control siRNA; siRPTPβ/ζ1, HUVEC transfected with siRPTPβ/ζ#1; siRPTPβ/ζ2, HUVEC transfected with si RPTPβ/ζ#2. The Western blot on top shows effective down-regulation of RPTPβ/ζ by both siRNA sequences used.

Since RPTPβ/ζ affects VEGF_165_-induced signaling related to cell migration, we tested whether it also affects VEGF_165_-induced endothelial cell migration. Down-regulation of RPTPβ/ζ expression by siRNA abolished VEGF_165_-induced HUVEC migration (Figure 4), highlighting a role for RPTPβ/ζ in the angiogenic effects of VEGF_165_.

### VEGF directly interacts with RPTPβ/ζ

**Figure 5:**
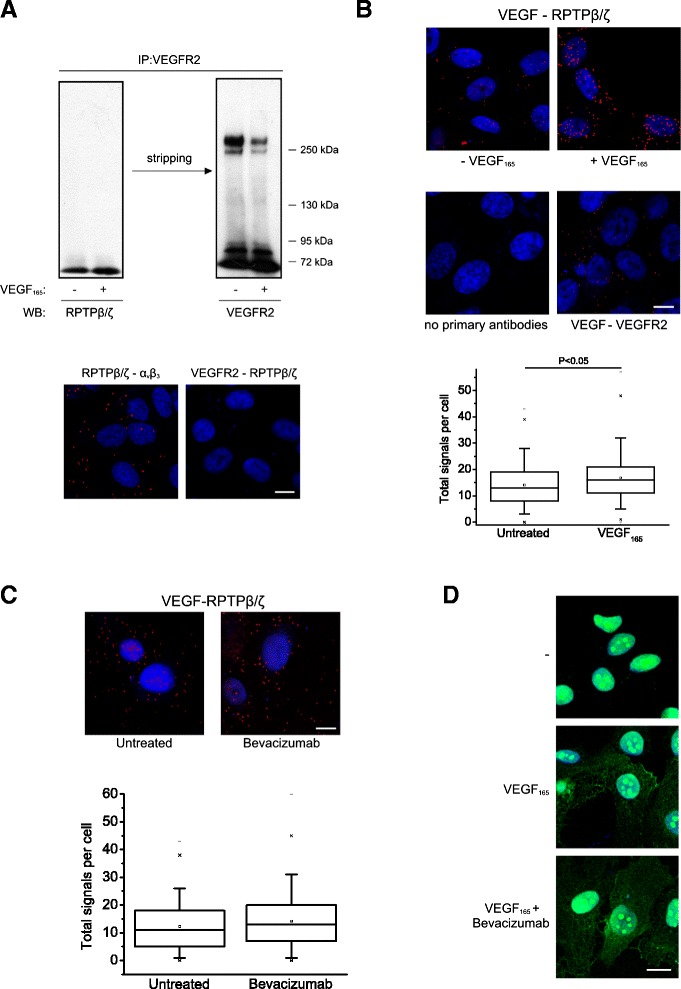
**VEGF directly interacts with RPTPβ/ζ in a VEGFR-independent manner. (A)** Serum-starved untreated or VEGF_165_-stimulated HUVEC lysates were immunoprecipitated for VEGFR2 and analyzed by Western blot for the presence of RPTPβ/ζ or VEGFR2 (up). No direct interaction between VEGFR2 with RPTPβ/ζ was observed by performing *in situ* PLA in HUVEC (down). The RPTPβ/ζ-ανβ_3_ interaction was used as a positive control. Data are from two independent experiments. **(B)** Formation of VEGF-RPTPβ/ζ complexes as evidenced by *in situ* PLA in HUVEC, untreated or after addition of exogenous VEGF_165_ (10 ng/ml) at 24 h. The VEGF-VEGFR2 interaction was used as a positive control. Data are from five independent experiments. **(C)** Formation of VEGF-RPTPβ/ζ complexes as evidenced by *in situ* PLA in HUVEC in the absence or the presence of bevacizumab (250 μg/ml). The box plots in **B** and **C** indicate the median, mean and range of the detected signals (n > 20 image fields with ~4 cells per image per sample type, each sample run at least in duplicate) from three independent experiments. **(D)** Immunofluorescence images stained for NCL (green) and nucleus (blue) in serum starved HUVEC treated for 5 h at 37°C with VEGF_165_ (10 ng/ml) in the presence or the absence of bevacizumab (250 μg/ml). Representative pictures from three independent experiments. Scale bars in all cases correspond to 10 μm.

It has been previously shown that RPTPβ/ζ interacts with both α_ν_β_3_ and NCL in HUVEC [8,11], both of which are involved in VEGF_165_-induced endothelial cell migration [2,3,13,14]. Since VEGFR2 is also required for VEGF_165_-induced endothelial cell migration [1], we tested whether RPTPβ/ζ interacts with VEGFR2. By performing immunoprecipitation/Western blot and PLA assays, we found that VEGFR2 does not associate with RPTPβ/ζ (Figure 5A). We then tested the possibility that VEGF directly associates with RPTPβ/ζ by performing PLA assays in HUVEC, which express endogenous levels of VEGF (Additional file 2). As shown in Figure 5B, endogenous VEGF formed complexes with RPTPβ/ζ, suggesting a direct interaction between the two molecules. Interestingly, addition of exogenous VEGF_165_ to HUVEC, which led to increased VEGF immunostaining (Additional file 2), increased VEGF-RPTPβ/ζ PLA signals (Figure 5B), verifying the specificity of the signal. In these assays, the interaction of VEGF with VEGFR2 was used as positive control. Interaction of VEGF with RPTPβ/ζ was also observed in human glioblastoma U87MG cells, by using a combination of immunoprecipitation/Western blot, double immunofluorescence and PLA assays. These cells express higher levels of endogenous VEGF compared with HUVEC and the PLA signals showing interaction with RPTPβ/ζ were also higher than those observed in HUVEC (Additional file 2). Interestingly, VEGF was not found to interact with α_ν_β_3_ (Additional file 3).

Bevacizumab used at a concentration that inhibits VEGF-induced cell migration and binding to both VEGFR1 and VEGFR2 [23], did not affect interaction of VEGF with RPTPβ/ζ (Figure 5C), suggesting that this interaction involves a different region on the VEGF molecule than the VEGFR binding site. Supporting this notion, bevacizumab did not inhibit VEGF_165_-induced cell surface NCL localization (Figure 5D), which is also mediated by RPTPβ/ζ as discussed above. Bevacizumab at the same concentration significantly inhibited interaction of VEGF with VEGFR2 (Additional file 4). These data clearly indicate that although binding to VEGFR2 is essential for VEGF-induced endothelial cell migration [2], some of the angiogenic actions of VEGF, such as cell surface NCL localization, are not inhibited by bevacizumab. Taking into account that angiogenic factors, such as PTN, hepatocyte growth factor and even VEGF itself act through cell surface NCL [12], the lack of effect of bevacizumab on VEGF_165_-induced cell surface NCL localization may explain at least some of the cases of resistance development to bevacizumab, e.g. in glioblastomas, where classical VEGF signaling through VEGFRs has been found to remain inhibited [24]. Moreover, our data provide a mechanistic support to the notion that RPTPβ/ζ is a valuable target for glioblastoma therapies [25,26].

There are two transmembrane isoforms of RPTPβ/ζ, the long isoform that has been described as a CS proteoglycan, and the short isoform considered a glycoprotein. Up to date, it is still unclear which of the two RPTPβ/ζ isoforms are responsible for each of its actions and it is not known whether glycosylation of these isoforms differs among different types of cells. Efforts to identify its CS glycanation suggest that it is regulated both developmentally and in different pathophysiological situations [27] and our unpublished observations based on Western blot analyses of RPTPβ/ζ expressed in different cell types support the notion of a cell-type specific RPTPβ/ζ glycosylation. PTN binding to RPTPβ/ζ involves both low (*K*_*d*_ = 3 nM) and high (*K*_*d*_ = 0.25 nM) affinity binding sites [28], which represent more than one sites of interaction that involve both the protein core and the CS chains of the receptor [5]. Oversulfation of CS is essential for PTN affinity and PTN-mediated functions [29-32], which is in line with our observation that CS-E inhibits interaction of PTN with RPTPβ/ζ in both HUVEC and U87MG cells (Additional file 5). It should be noted, however, that although inhibition in U87MG cells was at the level of 70% (total signals per cell in control: 23 ± 7 and in CS-E treated cells: 8 ± 1), in HUVEC it was smaller, at the level of 50% (total signals per cell in control: 6 ± 0.4 and in CS-E treated cells: 3 ± 0.3).

**Figure 6:**
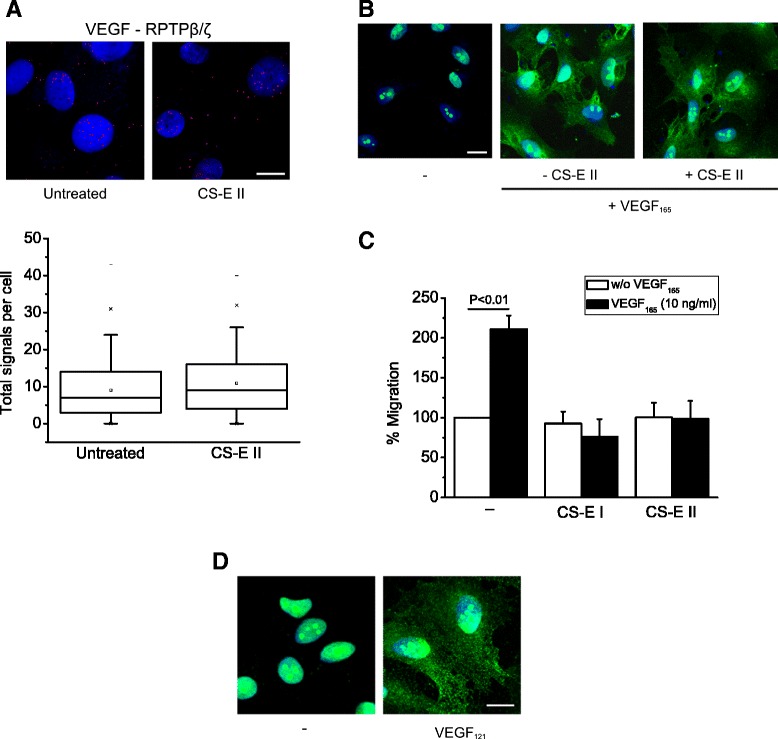
**Effect of CS-E on VEGF**_**165**_ **-induced endothelial cell signaling and migration. (A)** Formation of VEGF-RPTPβ/ζ complexes as evidenced by *in situ* PLA in HUVEC in the absence or the presence of CS-E II (100 ng/ml). The box plots indicate the median, mean and range of the detected signals (n > 20 image fields with ~4 cells per image per sample type, each sample run at least in duplicate) from three independent experiments. **(B)** Immunofluorescence images stained for NCL (green) and nucleus (blue) in serum starved HUVEC treated for 5 h at 37°C with VEGF_165_ (10 ng/ml) in the absence or the presence of CS-E II (100 ng/ml). Representative pictures from two independent experiments. **(C)** Effect of CS-E I and II (both at 100 ng/ml) on VEGF_165_-induced HUVEC migration. Data are from three independent experiments and are expressed as mean ± s.e.m. percentage change in number of migrating cells compared with the non stimulated untransfected cells (set as default 100). **(D)** Immunofluorescence images stained for NCL (green) and nucleus (blue) in serum starved HUVEC treated for 5 h at 37°C with VEGF_121_ (10 ng/ml). Representative pictures from two independent experiments. Scale bars in all cases correspond to 10 μm.

Since VEGF has been shown to be able to bind to CS-E similarly to heparan sulphate, but does not bind CS-C or CS-A [33], we investigated whether CS-Ε also inhibits VEGF interaction with RPTPβ/ζ. CS-E decreased interaction of VEGF with RPTPβ/ζ in U87MG cells (Additional file 6), but had no effect on HUVEC (Figure 6A), in line with the observation that it did not affect VEGF_165_-induced cell surface NCL localization (Figure 6B). This difference could be explained by the hypothesis that cell surface NCL localization depends on the interaction of VEGF with the short, non-proteoglycan isoform of RPTPβ/ζ. Interestingly and in favor of such a possibility, in U87MG cells that express higher amounts of endogenous VEGF than HUVEC, we performed IP/Western blot assays and observed that CS-E inhibits interaction of both PTN and VEGF with the long, but not the short RPTPβ/ζ isoform (Additional file 6). These data suggest that interaction of VEGF (and PTN) with the short RPTPβ/ζ isoform may not involve CS-E chains. On the other hand, CS-E abolished VEGF_165_-induced HUVEC migration (Figure 6C), an effect that may involve a different cell surface molecule or parallel signaling pathways that are important for cell migration. This point is under further investigation.

The idea that the interaction of VEGF with RPTPβ/ζ does not involve its heparin-binding properties is further supported by the observation that VEGF_121_, which induces HUVEC migration but does not contain the heparin-binding site of VEGF [34], also induces cell surface NCL localization (Figure 6D). Collectively, our data suggest that interaction of VEGF with RPTPβ/ζ may involve a part of the molecule that is distinct from those involved in VEGFR or glycosaminoglycan binding. This notion is similar to the recent finding that the high affinity neuropilin-1 binding of VEGF-A involves the exon 8-encoded C-terminal Arg [35]. Tuftsin, a naturally occurring TKPR peptide with sequence similarity to the sequence coded by exon 8 of VEGF, blocks VEGF_165_-induced autophosphorylation of VEGFR2, without inhibiting VEGF binding to VEGFR2 [36]. Taking into account that PTN binding to RPTPβ/ζ involves PTN's carboxy terminal region that is rich in basic amino acids [37], and that PTN inhibits VEGF binding to RPTPβ/ζ (see below), one can speculate that binding of VEGF to RPTPβ/ζ might be mediated by its exon 8-encoded sequence.

**Figure 7:**
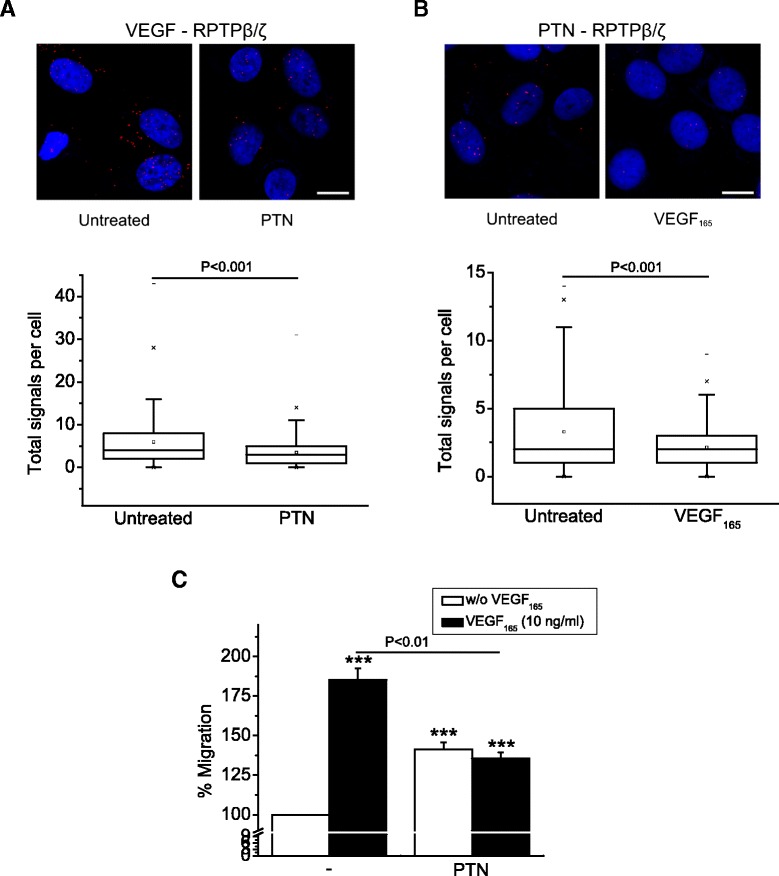
**PTN and VEGF compete for binding to RPTPβ/ζ. (A)** Formation of VEGF-RPTPβ/ζ complexes as evidenced by *in situ* PLA in HUVEC in the absence or the presence of exogenous PTN (100 ng/ml). **(Β)** Formation of PTN-RPTPβ/ζ complexes as evidenced by *in situ* PLA in HUVEC in the absence or the presence of exogenous VEGF_165_ (10 ng/ml). The box plots in **A** and **B** indicate the median, mean and range of the detected signals (n > 20 image fields with ~4 cells per image per sample type, each sample run at least in duplicate) from three independent experiments in each case. **(C)** Effect of PTN (100 ng/ml) on VEGF_165_-induced HUVEC migration. Data are from five independent experiments and are expressed as mean ± s.e.m. percentage change in number of migrating cells compared with the non stimulated untransfected cells (control, set as default 100). Asterisks denote a statistically significant difference from control. ***P < 0.001.

Finally, we found that the interaction of VEGF with RPTPβ/ζ was decreased in the presence of PTN in both HUVEC (Figure 7A) and U87MG cells (Additional file 7), suggesting that these two growth factors share a common binding site on RPTPβ/ζ. This is further supported by the observation that the PTN-RPTPβ/ζ interaction was also decreased in the presence of exogenous VEGF_165_ (Figure 7B). It has been previously suggested that besides the CS chains, the core RPTPβ/ζ protein is also involved in binding to PTN [28,38], which may also be the site of interaction of the short RPTPβ/ζ transmembrane isoform with VEGF, as discussed above.

PTN stimulates human endothelial cell migration by approximately 40% [6,8,11], an effect that is smaller than that of other growth factors, such as VEGF. Interestingly, PTN decreased VEGF_165_-induced HUVEC migration to the levels of its own, smaller stimulatory effect (Figure 7C), in agreement with a previous study showing that PTN did not abolish but decreased VEGF_165_-induced endothelial cell infiltration of matrigel *in vivo* to the levels of its own stimulation [39]. These data favor the notion that PTN and VEGF compete for binding to RPTPβ/ζ, as discussed above, and highlight a possible role for PTN as a regulator of angiogenesis, by limiting the aberrant effect of VEGF, while it still induces a smaller, significant stimulatory effect.

It is important to note that although RPTPβ/ζ is required for cell migration induced by PTN [6] and VEGF_165_ (present study), it is not sufficient by itself to induce cell migration, based on our previous data showing that midkine [8] or PTN_112–136_ [40], which lead to c-Src Tyr530 dephosphorylation and β_3_ Τyr773 phosphorylation through RPTPβ/ζ, do not induce endothelial cell migration. It seems that other receptors/signaling pathways are activated in parallel with the RPTPβ/ζ/c-Src/α_ν_β_3_ pathway to complementarily induce cell migration. Such a pathway for VEGF_165_ involves at least VEGFR2 and the cross-talk of signaling molecules activated by VEGF_165_ through RPTPβ/ζ, α_ν_β_3_ and VEGFR2 or other cell surface binding molecules in different cell types is under further investigation.

## Conclusion

**Figure 8:**
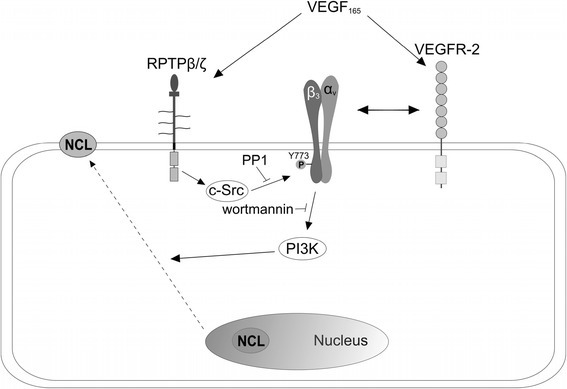
**Schematic representation of the proposed mechanism that involves RPTPβ/ζ and leads to increased α**_**ν**_ **β**_**3**_ **-VEGFR2 interaction, cell surface NCL localization and stimulation of cell migration by VEGF.** Binding of VEGF_165_ to RPTPβ/ζ on the surface of endothelial or cancer cells leads to c-Src activation, β_3_ Tyr773 phosphorylation and increased interaction of α_ν_β_3_ with VEGFR2, as well as PI3K activation and translocation of NCL from the nucleus to the cell membrane. Both are required for VEGF_165_-induced endothelial cell migration. For more details see text.

RPTPβ/ζ is a receptor-type protein tyrosine phosphatase expressed in several types of cancer and involved in cell migration, cancer progression and metastasis [5,27]. The current study identifies RPTPβ/ζ as a novel cell membrane binding molecule for VEGF_165_, which regulates c-Src-mediated β_3_ Tyr773 phosphorylation and interaction with VEGFR2, cell surface NCL localization through PI3K activation and endothelial cell migration (Figure 8). These data are of high significance, especially taking into account that the interaction of VEGF with RPTPβ/ζ and the downstream angiogenic VEGF actions, such as cell surface NCL localization, do not seem to be affected by existing anti-VEGF drugs, such as bevacizumab, and may explain the resistance developed by tumor types, e.g. glioblastoma, in such therapies [24,41]. They thus warrant exploitation of RPTPβ/ζ for the possible development of alternative or additive anti-angiogenic therapies, especially in cases where resistance develops.

## Methods

### Materials

Human recombinant VEGF_165_ was prepared as previously described [39]. VEGF_121_ was purchased from RELIATech GmbH (Wolfenbüttel, Germany). Bevacizumab (AVASTIN) was from Roche Applied Science (Indianapolis, IN, USA). Human recombinant PTN was from PeproTech, Inc. (Rocky Hill, NJ, USA) or prepared as previously described [39]. PTN from both sources was equally active in all cases. CS-E was either from Seikagaku Corporation (Tokyo, Japan) (CS-E I), or prepared from a crude preparation of squid cartilage CS (purchased from Yantai Changsen Chemical Co., Ltd., Shandong, China) (CS-E II), both sources being equally active. PP1, wortmannin and U0126 were from TOCRIS (Minneapolis, MN, USA) and SU1498 was from Santa Cruz Biotechnology Inc. (Santa Cruz, CA, USA). All secondary horseradish peroxidase-conjugated antibodies were from Cell Signaling Technology Inc. (Beverly, MA, USA). Human IgG and other reagents were from Sigma (St. Louis, MO, USA).

### Cell culture

HUVEC, human glioma U87MG cells and CHO cells expressing α_ν_ but deficient in endogenous β_3_ integrin or stably transfected to express wild type β_3_, β_3_Y773F, β_3_Y785F or β_3_Y773F/Y785F were cultured as previously described [8,11]. Cell culture reagents were from BiochromKG (Seromed, Germany). All cultures were maintained at 37°C, 5% CO_2_, and 100% humidity. When cells reached 70-80% confluence, they were serum starved for 16 h (where indicated) before performing migration assays, lysed for immunoprecipitation/Western blot assays or fixed for immunofluorescence assays and *in situ* PLA.

### Immunofluorescence

Cells were fixed with 4% formaldehyde in phosphate-buffered saline (PBS) pH 7.4 for 10 min and permeabilized with 0.1% Triton in PBS for 15 min. After being washed 3 times with PBS, the cells were blocked with PBS containing 3% BSA and 10% fetal bovine serum (FBS) for 1 h at room temperature. Cells were stained with the following primary antibodies: rabbit anti-VEGF (1:250; Santa Cruz Biotechnology Inc.), mouse anti-RPTPβ/ζ (1:250; BD Biosciences, San Diego, CA, USA), mouse anti-α_ν_β_3_ (1:500; Merck Millipore, Darmstadt, Germany), and rabbit anti-NCL (1:1,000, Sigma). Cells were then incubated with fluorescent Alexa secondary antibodies (1:500; Molecular Probes, Carlsbad, CA, USA). Nuclei were stained with Draq5 (Biostatus Limited, Leicestershire, UK). Cells were mounted with Mowiol 4–88 (Merck Millipore) and visualized at room temperature with Leica SP5 (X63 objective with a numerical aperture of 1.4) confocal microscope.

### *In situ* PLA

For detection of protein-protein interactions, *in situ* PLA was performed. The components used (Sigma) were as follows: anti-mouse PLA plus probe, anti-rabbit PLA minus probe and Detection Reagents Orange. HUVEC or U87MG cells were grown on μ-Chamber 12 well on glass slides (Ibidi©, Martinsried, Germany). After reaching 80% confluence or after appropriate treatment of cells, the assay was performed according to the manufacturer’s instructions. Briefly, after fixation and blocking, cells were incubated with the primary antibodies: mouse anti-VEGF (1:250), rabbit anti-VEGF (1:250), rabbit anti-Flk-1 (1:250), mouse anti-Flk-1 (1:250), mouse anti-NCL (1:50), goat anti-RPTPβ/ζ (1:250) (all from Santa Cruz Biotechnology Inc.), mouse anti-α_ν_β_3_ (1:500; Merck Millipore), mouse anti-PTN (1:500; Abnova, Heidelberg, Germany) and mouse anti-RPTPβ/ζ (1:250; BD Biosciences). Subsequently, cells were incubated with secondary antibodies conjugated with oligonucleotides. After hybridization and ligation of the oligonucleotides, the DNA was amplified. A detection mixture detected the amplicons, resulting in red fluorescence signals. Nuclei were counterstained with Draq5; cells were mounted with Mowiol 4–88 and visualized at room temperature with Leica SP5 confocal microscope.

### Quantification of *in situ* PLA signals

Estimation of nuclei and cytoplasm size was performed using the Duolink ImageTool software (Olink Bioscience). In order to calculate the total number of spots per cell, an algorithmic procedure was developed and implemented in the Matlab environment (The MathWorks Inc., Massachusetts, USA). “I” denotes an immunofluorescence image of m × n size. Such an image can be seen as a “stack” of three matrices of the same size; representing the red, green and blue values for each pixel indicated as IR, IG, IB for the three color bands, respectively. For every pixel at position (i, j) correspond three values; IR(i, j), IG(i, j), IB(i, j), ranging from 0 to 255. Image pre-processing was performed to identify pixels of red intensities less than a determined threshold value θ, in order to exclude background noise. Several values were tested and evaluated and finally θ was set to 50. In order to detect a red dot, the algorithm searches the red band (i.e., the IR matrix) for a group of at least c connected pixels. The value of the parameter c, which controls the minimum number of pixels that constitute a dot, c was set to 10. Moreover, in the case of overlapping dots, large groups of pixels were identified. In such cases, the true number of dots was calculated as [n /100] since a typical dot contains approximately 100 pixels, where n is the total number of pixels of the initial group and the operator [α] is used to round the number α into its nearest integer.

### RNA interference

Cells were grown to 50% confluence in medium without antibiotics. Transfection was performed in serum-free medium for 4 h using annealed RNA for RPTPβ/ζ (siRPTPβ/ζ#1, VBC Biotech Services, Vienna, Austria), as previously described [6]. Another siRNA sequence for RPTPβ/ζ (siRPTPβ/ζ#2, Hs_PTPRZ1_1 FlexiTube siRNA, Qiagen GmbH, Germany) was also used as a control for off-target effects. JetSI-ENDO (Polyplus Transfection, Illkirch, France) or Lipofectamine® RNAiMAX (Life Technologies) were used as transfection reagents. Double-stranded negative control siRNA (Ambion, Austin, TX, USA) was used in all experiments. Cells were incubated for another 48 h in serum-containing medium and lysed, serum starved or fixed before further experiments.

### Migration assays

Migration assays were performed as previously described [8,11] in 24-well microchemotaxis chambers (Corning, Inc., Lowell, MA, USA) using uncoated polycarbonate membranes with 8 μm pores. Serum-starved cells were harvested, resuspended at a concentration of 10^5^ cells/0.1 ml in serum-free medium containing 0.25% bovine serum albumin (BSA) and loaded in the upper chamber. The bottom chamber was filled with 0.6 ml of serum-free medium containing 0.25% BSA and the tested substances. Cells were incubated for 4 h at 37°C. After completion of the incubation, filters were fixed and stained with 0.33% toluidine blue solution. The cells that migrated through the filters were quantified by counting the entire area of each filter, using a grid and a microscope with a X20 objective (Optech Microscope Services Ltd., Thames, UK).

### PI3K p85 ELISA

The levels of total and phosphorylated PI3K p85 were quantified using Fast Activated Cell-based ELISA assays (Active Motif, Carlsbad, CA, USA) according to the manufacturer's instructions. Briefly, cells were cultured in 96-well plates one day prior to manipulation. Serum starved CHO cells or HUVEC were treated with 10 ng/ml VEGF_165_ for 10 min (in the presence or absence of inhibitors of signaling molecules where appropriate), fixed and incubated with anti–phospho and anti-total p85 antibodies.

### Immunoprecipitation assay

Cells were lysed with RIPA buffer, as previously described [8]. Three mg of total protein were incubated with primary antibody for 16 h at 4°C under continuous agitation. The primary antibodies used were: mouse anti-VEGF (3 μg), mouse anti-Flk-1 (3 μg), goat anti-RPTPβ/ζ (3 μg), goat anti-PTN (3 μg) (Santa Cruz Biotechnology Inc.) and goat anti-β_3_ (1.5 μg; Merck Millipore). Protein A- and protein G-agarose beads (Merck Millipore) were added, samples were further incubated for 2 h at 4°C, and beads with bound proteins were collected by centrifugation (5,000 *g* for 5 min at 4°C) and washed twice with ice-cold PBS pH 7.4. Immunoprecipitated proteins were resuspended in SDS loading buffer and analyzed by Western blot.

### Western blot analysis

Proteins were analyzed by SDS-PAGE and transferred to Immobilon P membranes. Blocking was performed by incubating the membranes with Tris-buffered saline (TBS) pH 7.4 with 0.05% Tween (TBS-T), containing either 5% nonfat dry milk or 3% BSA. Membranes were incubated with primary antibodies for 16 h at 4°C under continuous agitation, washed 3 times with TBS-T, and incubated with secondary antibodies for 1 h at room temperature. Primary antibodies used were mouse anti-Flk-1 (1:500), goat anti-β_3_ (1:500), mouse anti-VEGF (1:500), rabbit anti-phospho-β_3_(Y773) (1:1,000; Santa Cruz Biotechnology Inc.), mouse anti-RPTPβ/ζ (1:500; BD Biosciences), rabbit anti-c-Src (1:1,000; Merck Millipore), rabbit anti-phospho-c-Src(Y418) (1:1,000; Acris Antibodies GmbH) and rabbit anti-non-phospho-c-Src (1:1,000; Cell Signaling Technology Inc.). Detection of immunoreactive bands was performed using the enhanced chemiluminescence detection kit (Pierce Biotechnology, Rockford, IL, USA). Protein levels were quantified using the ImagePC image analysis software (Scion Corp., Frederick, MD, USA).

### Statistical analysis

Results are expressed as mean ± s.e.m. or by using box plots, where the box is determined by the 25^th^ and 75^th^ percentiles, the whiskers are determined by the 5^th^ and 95^th^ percentiles, the line in the box marks the median and the empty square in the box marks the mean. Outliers have been plotted as individual points. Where applicable, variability between the results from each group and the corresponding control was determined by unpaired *t* test.

## Additional files

Additional file 1:
**c-Src activation may be independent of VEGF-VEGFR2 interaction.** Serum starved HUVEC were treated with VEGF_165_ (10 ng/ml) for 10 min in the presence or absence of either the selective VEGFR2 tyrosine kinase inhibitor SU1498 (10 μΜ) or bevacizumab (250 μg/ml). Cell lysates were analyzed by Western blot for non Tyr530 phosphorylated (npc-Src), Tyr419 phosphorylated (pc-Src) and total (tc-Src) c-Src. Numbers in brackets denote the average-fold change of the ratio npc-Src:tc-Src or pc-Src:tc-Src respectively, compared with the corresponding non stimulated, untransfected cells (set as default 1) from three independent experiments. Additional file 2:
**VEGF-RPTPβ/ζ interaction in human glioma U87MG cells.** (A) Immunofluorescence images of HUVEC and U87MG cells stained for VEGF (green) and nucleus (blue). Representative pictures from three and two independent experiments respectively. (B) U87MG cell lysates were immunoprecipitated for RPTPβ/ζ or VEGF. Immunoprecipitates were analyzed by Western blot for the presence of VEGF or RPTPβ/ζ, respectively. IgG was used as a negative control. Representative blots from two independent experiments. (C) Immunofluorescence images of U87MG cells stained for VEGF (green), RPTPβ/ζ (red) and nucleus (blue). Representative pictures from two independent experiments. (D) *In situ* PLA signals were detected as red dots, indicating the direct formation of VEGF- RPTPβ/ζ complexes. Representative pictures from three independent experiments. Scale bars in A, C and D correspond to 10 μm. (E) Formation of VEGF-RPTPβ/ζ complexes as evidenced by *in situ* PLA in HUVEC and U87MG cells. The box plots indicate the median, mean and range of the detected signals (n > 20 image fields with 4-6 cells per image per sample type, each sample run in duplicate) from four independent experiments. Additional file 3:
**VEGF does not interact with α**_**ν**_ **β**_**3**_ **.** (A) Immunofluorescence images of U87MG cells cultured in serum-containing medium and stained for VEGF (green), α_ν_β_3_ (red) and nucleus (blue). Representative pictures from two independent experiments. (B) The absence of *in situ* PLA signals indicates lack of the VEGF-α_ν_β_3_ direct interaction in both HUVEC and U87MG cells. The PTN-α_ν_β_3_ interaction in both types of cells was used as a positive control. Representative pictures from two independent experiments. Additional file 4:
**Bevacizumab inhibits VEGF-VEGFR2 interaction.** Formation of VEGF-VEGFR2 complexes as evidenced by *in situ* PLA in HUVEC in the absence or the presence of bevacizumab (250 μg/ml). The box plots indicate the median, mean and range of the detected signals (n > 20 image fields with ~4 cells per image per sample type, each sample run at least in duplicate) from two independent experiments. Scale bars in all cases correspond to 10 μm. Additional file 5:
**CS-E inhibits PTN-RPTPβ/ζ interaction in both HUVEC and U87MG cells.** Formation of PTN-RPTPβ/ζ complexes as evidenced by *in situ* PLA in HUVEC (A) and U87MG cells (B) in the absence or presence of CS-E II (100 ng/ml). The box plots indicate the median, mean and range of the detected signals (n = 20 image fields with ~6 cells per image per sample type, each sample run in duplicate) from four (A) and three (B) independent experiments. Scale bars in all cases correspond to 10 μm. Additional file 6:
**CS-E inhibits VEGF-RPTPβ/ζ interaction in U87MG cells.** (A) Formation of VEGF-RPTPβ/ζ complexes as evidenced by *in situ* PLA in U87MG cells in the absence or presence of CS-E II (100 ng/ml). The box plots indicate the median, mean and range of the detected signals (n = 8 image fields with ~4 cells per image per sample type, each sample run in duplicate) from three independent experiments. Scale bar corresponds to 10 μm. (B) U87MG cell lysates were immunoprecipitated for VEGF. Immunoprecipitates were analyzed by Western blot for the presence of RPTPβ/ζ. (C) U87MG cell lysates were immunoprecipitated for PTN. Immunoprecipitates were analyzed by Western blot for the presence of RPTPβ/ζ. In B and C, representative blots from two independent experiments are shown. Additional file 7:
**PTN inhibits VEGF-RPTPβ/ζ interaction in U87MG cells.** (A) Formation of VEGF-RPTPβ/ζ complexes as evidenced by *in situ* PLA in U87MG cells in the absence or presence of PTN (100 ng/ml). The box plots indicate the median, mean and range of the detected signals (n = 6 image fields with ~ 5 cells per image per sample type, each sample run in duplicate). Scale bar corresponds to 10 μm. Data come from three independent experiments. (B) Cell lysates were immunoprecipitated for RPTPβ/ζ. Immunoprecipitates were analyzed by Western blot for the presence of VEGF. IgG was used as a negative control. Representative blots from two independent experiments.

## Abbreviations

BSA: Bovine serum albumin
CS: Chondroitin sulphate
ELISA: Enzyme-linked immunoassay
FBS: Fetal bovine serum
NCL: Nucleolin
PBS: Phosphate buffered saline
PI3K: Phosphatidylinositol 3-kinase
PLA: Proximity ligation assay
PTN: Pleiotrophin
RPTPβ/ζ: Receptor protein tyrosine phosphatase beta/zeta
TBS: Tris-buffered saline
VEGF: Vascular endothelial growth factor
VEGFR2: Vascular endothelial growth factor receptor 2
