# Supplementary material for: Receptor protein tyrosine phosphatase beta/zeta is a functional binding partner for vascular endothelial growth factor
Source: Mol Cancer. 2015 Feb 3;14(1):19. doi: 10.1186/s12943-015-0287-3 (PMC4323219; doi:10.1186/s12943-015-0287-3)
Supplement: Additional file 1: — c-Src activation may be independent of VEGF-VEGFR2 interaction. Serum starved HUVEC were treated with VEGF165 (10 ng/ml) for 10 min in the presence or absence of either the selective VEGFR2 tyrosine kinase inhibitor SU1498 (10 μΜ) or bevacizumab (250 μg/ml). Cell lysates were analyzed by Western blot for non Tyr530 phosphorylated (npc-Src), Tyr419 phosphorylated (pc-Src) and total (tc-Src) c-Src. Numbers in brackets denote the average-fold change of the ratio npc-Src:tc-Src or pc-Src:tc-Src respectively, compared with the corresponding non stimulated, untransfected cells (set as default 1) from three independent experiments. [file 12943_2015_287_MOESM1_ESM.pdf]

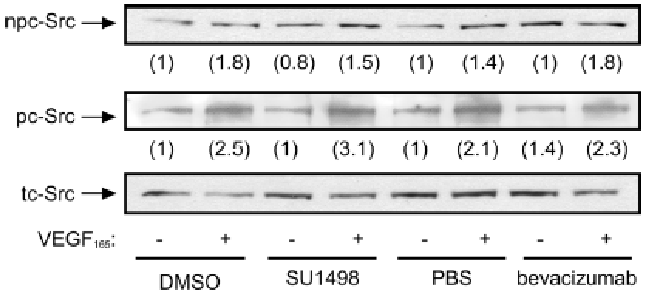

Additional file 1. C-Src activation may be independent of VEGF-VEGFR2 interaction. Serum starved HUVEC were treated with $VEGF_{165}$ (10 ng/ml) for 10 min in the presence or absence of either the selective VEGFR2 tyrosine kinase inhibitor SU1498 (10 μM) or bevacizumab (250 μg/ml). Cell lysates were analyzed by Western blot for non phosphorylated at Tyr530 (npc-Src), phosphorylated at Tyr419 (pc-Src) and total (tc-Src) c-Src. Numbers in brackets denote the average-fold change of the ratio npc-Src:tc-Src or pc-Src:tc-Src respectively, compared with the corresponding non stimulated, untransfected cells (set as default 1) from three independent experiments.
