# Supplementary material for: Receptor protein tyrosine phosphatase beta/zeta is a functional binding partner for vascular endothelial growth factor
Source: Mol Cancer. 2015 Feb 3;14(1):19. doi: 10.1186/s12943-015-0287-3 (PMC4323219; doi:10.1186/s12943-015-0287-3)
Supplement: Additional file 2: — VEGF-RPTPβ/ζ interaction in human glioma U87MG cells. (A) Immunofluorescence images of HUVEC and U87MG cells stained for VEGF (green) and nucleus (blue). Representative pictures from three and two independent experiments respectively. (B) U87MG cell lysates were immunoprecipitated for RPTPβ/ζ or VEGF. Immunoprecipitates were analyzed by Western blot for the presence of VEGF or RPTPβ/ζ, respectively. IgG was used as a negative control. Representative blots from two independent experiments. (C) Immunofluorescence images of U87MG cells stained for VEGF (green), RPTPβ/ζ (red) and nucleus (blue). Representative pictures from two independent experiments. (D) In situ PLA signals were detected as red dots, indicating the direct formation of VEGF- RPTPβ/ζ complexes. Representative pictures from three independent experiments. Scale bars in A, C and D correspond to 10 μm. (E) Formation of VEGF-RPTPβ/ζ complexes as evidenced by in situ PLA in HUVEC and U87MG cells. The box plots indicate the median, mean and range of the detected signals (n > 20 image fields with 4-6 cells per image per sample type, each sample run in duplicate) from four independent experiments. [file 12943_2015_287_MOESM2_ESM.pdf]

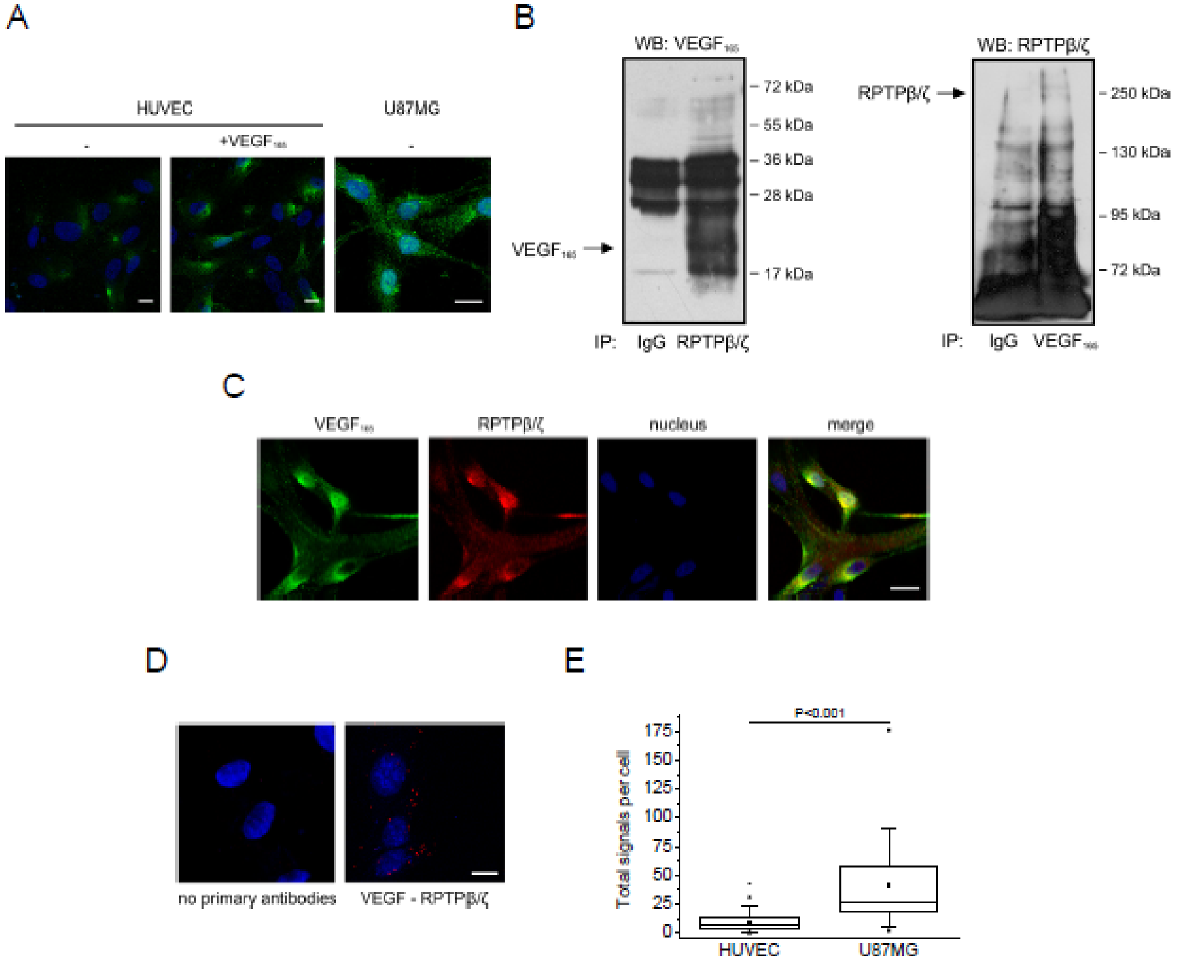

Additional file 2. VEGF-RPTPβ/ζ interaction in human glioma U87MG cells. (A) Immunofluorescence images of HUVEC and U87MG cells stained for $VEGF_{165}$ (green) and nucleus (blue). Representative pictures from three and two independent experiments respectively. **(B)** U87MG cell lysates were immunoprecipitated for RPTPβ/ζ or $VEGF_{165}$. Immunoprecipitates were analyzed by Western blot for the presence of $VEGF_{165}$ or RPTPβ/ζ, respectively. IgG was used as a negative control. Representative blots from two independent experiments. **(C)** Immunofluorescence images of U87MG cells stained for $VEGF_{165}$ (green), RPTPβ/ζ (red) and nucleus (blue). Representative pictures from two independent experiments. **(D)** *In situ* PLA signals were detected as red dots, indicating the direct formation of $VEGF_{165}$-RPTPβ/ζ complexes. Representative pictures from three independent experiments. Scale bars in A, C and D correspond to 10 μm. **(E)** Formation of VEGF-RPTPβ/ζ complexes as evidenced by *in situ* PLA in HUVEC and U87MG cells. The box plots indicate the median, mean and range of the detected signals ($n > 20$ image fields with 4-6 cells per image per sample type, each sample run in duplicate) from four independent experiments.
