# Supplementary material for: Receptor protein tyrosine phosphatase beta/zeta is a functional binding partner for vascular endothelial growth factor
Source: Mol Cancer. 2015 Feb 3;14(1):19. doi: 10.1186/s12943-015-0287-3 (PMC4323219; doi:10.1186/s12943-015-0287-3)
Supplement: Additional file 3: — VEGF does not interact with α ν β 3 . (A) Immunofluorescence images of U87MG cells cultured in serum-containing medium and stained for VEGF (green), ανβ3 (red) and nucleus (blue). Representative pictures from two independent experiments. (B) The absence of in situ PLA signals indicates lack of the VEGF-ανβ3 direct interaction in both HUVEC and U87MG cells. The PTN-ανβ3 interaction in both types of cells was used as a positive control. Representative pictures from two independent experiments. [file 12943_2015_287_MOESM3_ESM.pdf]

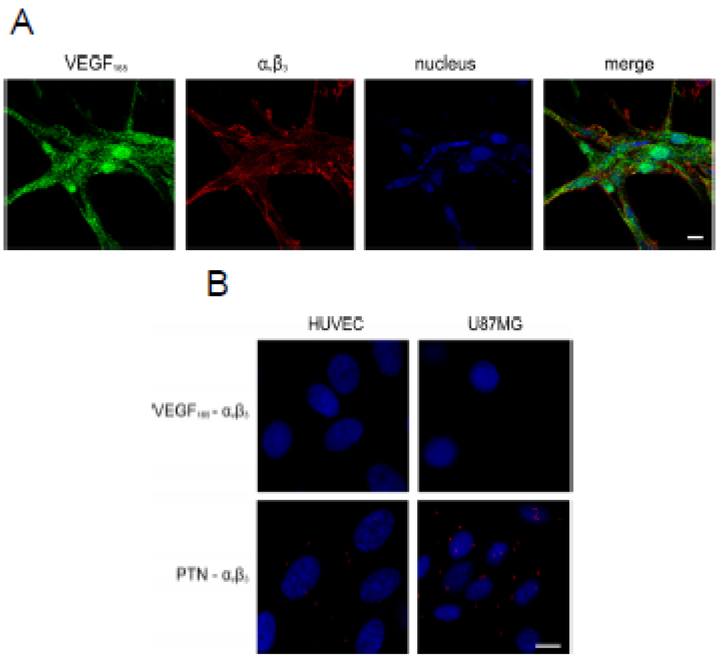

Additional file 3. $VEGF_{165}$ does not interact with $\alpha_v\beta_3$. (A) Immunofluorescence images of U87MG cells cultured in serum-containing medium and stained for $VEGF_{165}$ (green), $\alpha_v\beta_3$ (red) and nucleus (blue). Representative pictures from two independent experiments. **(B)** The absence of *in situ* PLA signals indicates lack of the $VEGF_{165}$-$\alpha_v\beta_3$ direct interaction in both HUVEC and U87MG cells. The PTN-$\alpha_v\beta_3$ interaction in both types of cells was used as a positive control. Representative pictures from two independent experiments.
