# Supplementary material for: Receptor protein tyrosine phosphatase beta/zeta is a functional binding partner for vascular endothelial growth factor
Source: Mol Cancer. 2015 Feb 3;14(1):19. doi: 10.1186/s12943-015-0287-3 (PMC4323219; doi:10.1186/s12943-015-0287-3)
Supplement: Additional file 4: — Bevacizumab inhibits VEGF-VEGFR2 interaction. Formation of VEGF-VEGFR2 complexes as evidenced by in situ PLA in HUVEC in the absence or the presence of bevacizumab (250 μg/ml). The box plots indicate the median, mean and range of the detected signals (n > 20 image fields with ~4 cells per image per sample type, each sample run at least in duplicate) from two independent experiments. Scale bars in all cases correspond to 10 μm. [file 12943_2015_287_MOESM4_ESM.pdf]

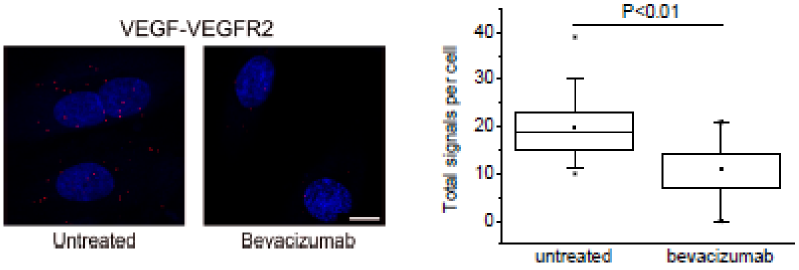

Additional file 4. Bevacizumab inhibits VEGF-VEGFR2 interaction. Formation of VEGF-VEGFR2 complexes as evidenced by *in situ* PLA in HUVEC in the absence or the presence of bevacizumab (250 µg/ml). The box plots indicate the median, mean and range of the detected signals (n > 20 image fields with ~4 cells per image per sample type, each sample run at least in duplicate) from two independent experiments. Scale bars in all cases correspond to 10 µm.
