# Supplementary material for: Receptor protein tyrosine phosphatase beta/zeta is a functional binding partner for vascular endothelial growth factor
Source: Mol Cancer. 2015 Feb 3;14(1):19. doi: 10.1186/s12943-015-0287-3 (PMC4323219; doi:10.1186/s12943-015-0287-3)
Supplement: Additional file 5: — CS-E inhibits PTN-RPTPβ/ζ interaction in both HUVEC and U87MG cells. Formation of PTN-RPTPβ/ζ complexes as evidenced by in situ PLA in HUVEC (A) and U87MG cells (B) in the absence or presence of CS-E II (100 ng/ml). The box plots indicate the median, mean and range of the detected signals (n = 20 image fields with ~6 cells per image per sample type, each sample run in duplicate) from four (A) and three (B) independent experiments. Scale bars in all cases correspond to 10 μm. [file 12943_2015_287_MOESM5_ESM.pdf]

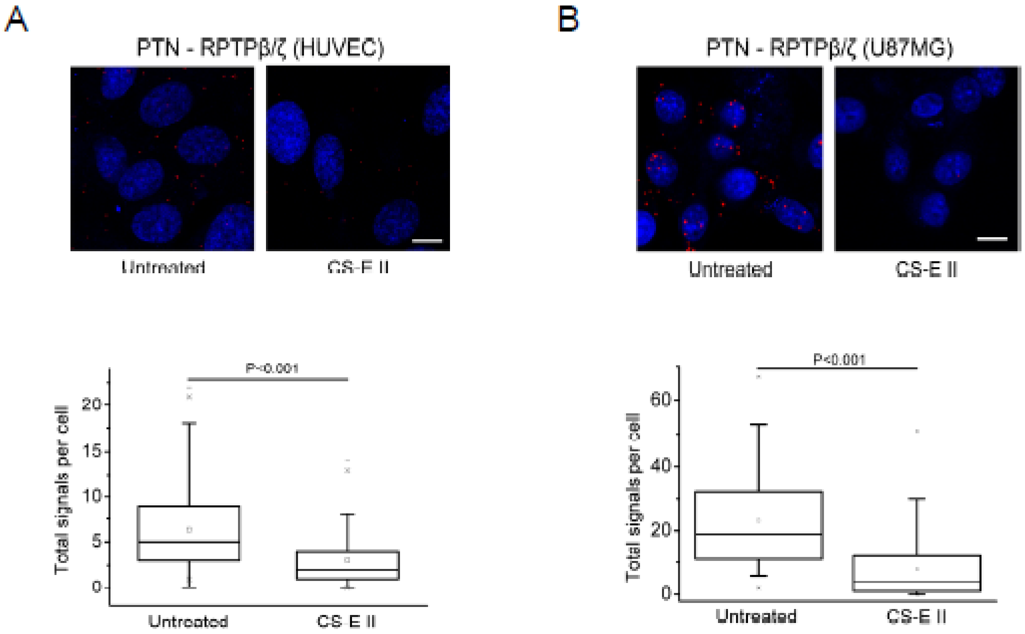

Additional file 5. CS-E inhibits PTN-RPTPβ/ζ interaction in both HUVEC and U87MG cells. Formation of PTN-RPTPβ/ζ complexes as evidenced by *in situ* PLA in HUVEC **(A)** and U87MG cells **(B)** in the absence or presence of CS-E II (100 ng/ml). The box plots indicate the median, mean and range of the detected signals (n = 20 image fields with ~6 cells per image per sample type, each sample run in duplicate) from four (A) and three (B) independent experiments. Scale bars in all cases correspond to 10 μm.
