# Supplementary material for: Receptor protein tyrosine phosphatase beta/zeta is a functional binding partner for vascular endothelial growth factor
Source: Mol Cancer. 2015 Feb 3;14(1):19. doi: 10.1186/s12943-015-0287-3 (PMC4323219; doi:10.1186/s12943-015-0287-3)
Supplement: Additional file 6: — CS-E inhibits VEGF-RPTPβ/ζ interaction in U87MG cells. (A) Formation of VEGF-RPTPβ/ζ complexes as evidenced by in situ PLA in U87MG cells in the absence or presence of CS-E II (100 ng/ml). The box plots indicate the median, mean and range of the detected signals (n = 8 image fields with ~4 cells per image per sample type, each sample run in duplicate) from three independent experiments. Scale bar corresponds to 10 μm. (B) U87MG cell lysates were immunoprecipitated for VEGF. Immunoprecipitates were analyzed by Western blot for the presence of RPTPβ/ζ. (C) U87MG cell lysates were immunoprecipitated for PTN. Immunoprecipitates were analyzed by Western blot for the presence of RPTPβ/ζ. In B and C, representative blots from two independent experiments are shown. [file 12943_2015_287_MOESM6_ESM.pdf]

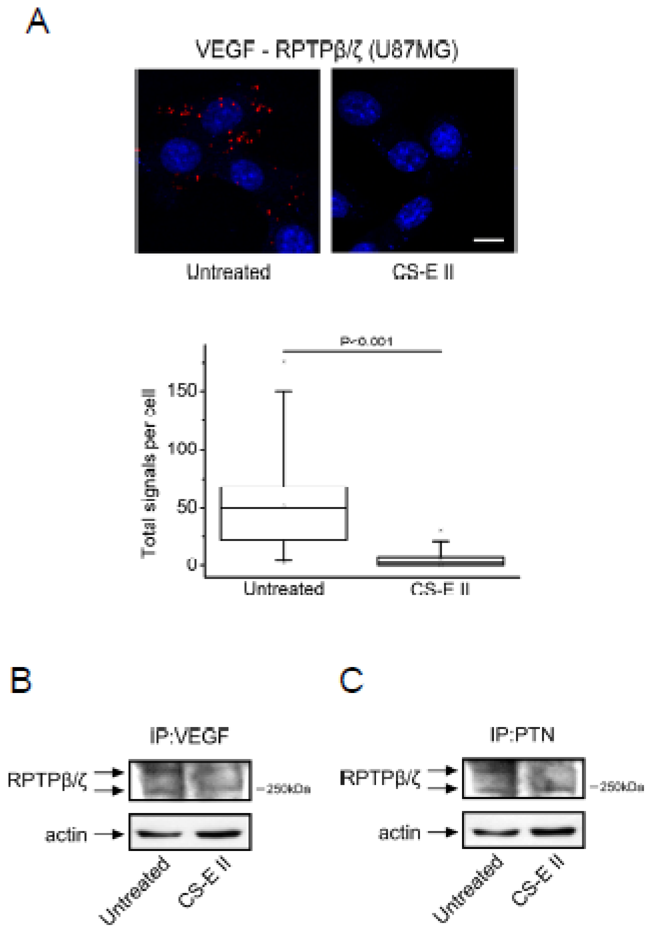

Additional file 6. CS-E inhibits VEGF-RPTPβ/ζ interaction in U87MG cells. (A) Formation of VEGF-RPTPβ/ζ complexes as evidenced by *in situ* PLA in U87MG cells in the absence or presence of CS-E II (100 ng/ml). The box plots indicate the median, mean and range of the detected signals (n = 8 image fields with ~4 cells per image per sample type, each sample run in duplicate) from three independent experiments. Scale bar corresponds to 10 μm. **(B)** U87MG cell lysates were immunoprecipitated for VEGF. Immunoprecipitates were analyzed by Western blot for the presence of RPTPβ/ζ. **(C)** U87MG cell lysates were immunoprecipitated for PTN. Immunoprecipitates were analyzed by Western blot for the presence of RPTPβ/ζ. In B and C, representative blots from two independent experiments are shown.
