# Supplementary material for: Receptor protein tyrosine phosphatase beta/zeta is a functional binding partner for vascular endothelial growth factor
Source: Mol Cancer. 2015 Feb 3;14(1):19. doi: 10.1186/s12943-015-0287-3 (PMC4323219; doi:10.1186/s12943-015-0287-3)
Supplement: Additional file 7: — PTN inhibits VEGF-RPTPβ/ζ interaction in U87MG cells. (A) Formation of VEGF-RPTPβ/ζ complexes as evidenced by in situ PLA in U87MG cells in the absence or presence of PTN (100 ng/ml). The box plots indicate the median, mean and range of the detected signals (n = 6 image fields with ~ 5 cells per image per sample type, each sample run in duplicate). Scale bar corresponds to 10 μm. Data come from three independent experiments. (B) Cell lysates were immunoprecipitated for RPTPβ/ζ. Immunoprecipitates were analyzed by Western blot for the presence of VEGF. IgG was used as a negative control. Representative blots from two independent experiments. [file 12943_2015_287_MOESM7_ESM.pdf]

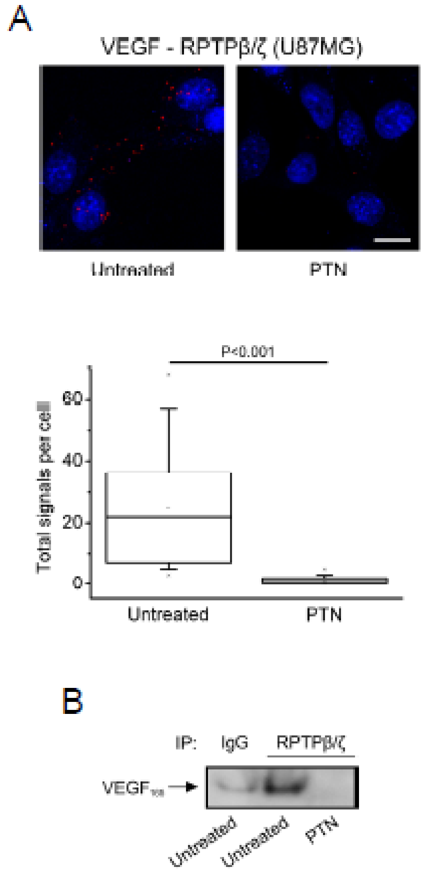

Additional file 7. PTN inhibits VEGF-RPTPβ/ζ interaction in U87MG cells. (A) Formation of VEGF-RPTPβ/ζ complexes as evidenced by *in situ* PLA in U87MG cells in the absence or presence of PTN (100 ng/ml). The box plots indicate the median, mean and range of the detected signals (n = 6 image fields with ~ 5 cells per image per sample type, each sample run in duplicate). Scale bar corresponds to 10 μm. Data come from three independent experiments. **(B)** Cell lysates were immunoprecipitated for RPTPβ/ζ. Immunoprecipitates were analyzed by Western blot for the presence of VEGF. IgG was used as a negative control. Representative blots from two independent experiments.
